# OLDER ADULT VOLUNTEERS AS MENTORS IN GERIATRIC EDUCATION

**DOI:** 10.1093/geroni/igad104.1576

**Published:** 2023-12-21

**Authors:** Janice Knebl

**Affiliations:** UNTHSC, Fort Worth, Texas, United States

## Abstract

Health professions education that involves older adults has been shown to be effective in improving students’ clinical skills and attitudes towards older adults. With the goal of introducing healthcare students to geriatric care through first-hand experiences, the “Seniors Assisting in Geriatrics Education (SAGE)” Program involves older adult volunteers age 65+ in virtual person-centered health promotion visits with teams of three to four students. Through an application process, mentors are selected and receive orientation materials before visits with their assigned team. Students represent eight disciplines: dietetics, medicine, nursing, pharmacy, physical therapy, physician assistant studies, social work, and speech language pathology. Students practice basic clinical skills and screening techniques while working as a team and receiving feedback and input from their mentor. In 2022, we surveyed older adult volunteers to evaluate their mentor experience using open-ended and Likert style questions on a scale from strongly agree to strongly disagree. Survey responses (n=99) show that most mentors agreed students collaborated well (94%), health promotion topics for each visit were beneficial (83% to 85%), and they were satisfied with their SAGE experience (96%). Mentors identified opportunities for improvement related to student and mentor preparation, in-person versus online visits, and establishing rapport between mentors and teams. Results were used to enhance mentor training and engagement and to create community partnerships for recruitment and mentor diversity. By prioritizing recruitment of and training for volunteers, older adults can actively and effectively participate in meaningful educational experiences with health professions students.

